# Brexpiprazole treatment for agitation in Alzheimer's dementia: A randomized study

**DOI:** 10.1002/alz.14282

**Published:** 2024-10-06

**Authors:** Yu Nakamura, Jun Adachi, Naoki Hirota, Katsuhiro Iba, Koichi Shimizu, Masami Nakai, Kaneyoshi Takahashi, Naoki Mori

**Affiliations:** ^1^ Faculty of Medicine/Graduate School of Medicine Kagawa University Kita‐gun Kagawa Japan; ^2^ Headquarters of Clinical Development Otsuka Pharmaceutical Co., Ltd Minato‐ku Tokyo Japan; ^3^ Headquarters of Clinical Development Otsuka Pharmaceutical Co., Ltd Chuo‐ku Osaka Japan; ^4^ Department of Medical Affairs Otsuka Pharmaceutical Co., Ltd Minato‐ku Tokyo Japan; ^5^ Department of Medical Affairs Otsuka Pharmaceutical Co., Ltd Chuo‐ku Osaka Japan

**Keywords:** Alzheimer's disease, brexpiprazole, efficacy, Japan, safety

## Abstract

**INTRODUCTION:**

We evaluated the efficacy and safety of brexpiprazole for the treatment of agitation in Alzheimer's dementia (AAD) in Japanese patients.

**METHODS:**

This was a phase 2/3 multicenter, randomized, double‐blind, placebo‐controlled, parallel‐group study. Patients with AAD were randomized to receive brexpiprazole 1 mg/day or 2 mg/day, or placebo (3:4:4) for 10 weeks.

**RESULTS:**

For the primary endpoint (change in Cohen‐Mansfield Agitation Inventory [CMAI] total score from baseline to Week 10), both brexpiprazole 1 mg and 2 mg groups demonstrated statistically significant improvement versus placebo (2 mg: least squares [LS] mean difference –7.2 [95% confidence interval (CI): –10.0 to –4.3], *p*‐value < 0.0001, 1 mg: LS mean difference –3.7 [95% CI: –6.8 to –0.7], *p*‐value = 0.0175). The incidences of treatment‐emergent adverse events reported in the brexpiprazole 1 mg, 2 mg, and placebo groups were 76.8%, 84.6%, and 73.8%, respectively.

**DISCUSSION:**

Brexpiprazole 1 mg/day and 2 mg/day for 10 weeks was efficacious and well tolerated.

**Highlights:**

- Brexpiprazole treatment for 10 weeks improved agitation in Alzheimer's dementia.
- The efficacy of brexpiprazole 1 mg/day has been confirmed for the first time.
- The incidence of adverse events was higher compared to the previous studies.
- Both brexpiprazole 1 mg/day and 2 mg/day were generally well tolerated.

## 1 BACKGROUND

Agitation is a common neuropsychiatric symptom observed in 45% of patients with Alzheimer's disease/dementia.^1^ The symptoms of agitation are characterized by excessive motor activity, verbal aggression, or physical aggression, which cause excess disability and are not attributable solely to another disorder (psychiatric, medical, or substance‐related).^2^ Agitation is not only related to a decline of cognitive function, impairment of activities in daily living and function, progression to severe Alzheimer's dementia, and death,^3^, ^4^, ^5^ but also to the burden on caregivers, increase in nursing and observation time, earlier admission to care facility, and rise in health care costs,^3^, ^6^, ^7^, ^8^, ^9^, ^10^, ^11^ indicating increased burden for patients, caregivers, and health care resources.

In one of the most aging countries, Japan,^12^ the prevalence of dementia in people ≥65 years of age is projected to exceed 25% nationwide by 2045.^13^ In line with this trend, the number of patients with Alzheimer's dementia in the United States is expected to be more than doubled by mid‐century, mainly fueled by aging of the “Baby Boomer” generation.^14^ Given also that the prevalence of dementia globally is estimated to more than double by 2050, the prevalence of agitation in Alzheimer's dementia (AAD) is also expected to rise significantly. As of 2023, brexpiprazole became the first and only US Food and Drug Administration (FDA)–approved treatment for agitation associated with dementia due to Alzheimer's disease, and in 2024, brexpiprazole was approved for the same indication in Canada and the Philippines. However, in many countries, there is no authority‐approved drug for AAD, resulting in off‐label use with treatments that have limited evidence‐based support for their use despite unfavorable benefit‐risk profiles.^15^, ^16^ Therefore, further accumulation of clinical knowledge in Japan will contribute to the global knowledge of the evolving brexpiprazole clinical profile.

AAD is associated with abnormalities in the important brain regions including frontal cortex and amygdala, in association with a defect of the serotonergic system, the relative preservation of dopaminergic function, and hyperreactivity of noradrenergic neurons.^17^, ^18^ Brexpiprazole acts as a partial agonist at serotonin 5‐hydroxytryptamine (HT)_1A_ and dopamine D_2_ receptors, and as an antagonist at serotonin 5‐HT_2A_ and noradrenaline α_1B_/α_2C_ receptors.^19^ Although the details of brexpiprazole's mechanism of action is still unclear, the alleviation of AAD symptoms is expected in response to these multiple actions. In patients with AAD, three global confirmation studies were conducted to evaluate the change in the Cohen‐Mansfield Agitation Inventory (CMAI) total score from baseline to Week 12 as a primary endpoint (ClinicalTrials.gov Identifier: NCT01862640, NCT01922258, and NCT03548584).^20^, ^21^ The first study had a fixed‐dose design, 1 mg/day or 2 mg/day, and brexpiprazole 2 mg/day demonstrated statistically significant improvement versus placebo. The second study had a flexible‐dose design, and brexpiprazole 0.5–2 mg/day showed numerical improvements over placebo. The third study had a fixed‐dose design, 2 mg or 3 mg/day, and the overall brexpiprazole group demonstrated statistically significant improvement versus the placebo group. In all three studies, brexpiprazole was well tolerated.

However, these prior studies were conducted in the United States and Europe and had very low representation of Asian populations. Considering this situation, we thought it important to confirm if brexpiprazole treatment is efficacious and well tolerated in non‐White populations as well. Therefore, we conducted the first confirmation study of brexpiprazole indicated for the treatment of AAD in Asian patients—in this case, Japanese patients—to evaluate the efficacy, safety, and tolerability of brexpiprazole 1 mg or 2 mg/day and explore the optimal dose. Brexpiprazole 2 mg/day was applied based on the results of the previously conducted global studies, and 1 mg/day was set to investigate the dose–response relationship in Japanese patients.

**RESEARCH IN CONTEXT**

1. **Systematic review**: For the treatment of agitation in Alzheimer's dementia, brexpiprazole is not approved in many countries, and the clinical profile of brexpiprazole is still evolving globally. Following the global studies to evaluate the efficacy and safety of brexpiprazole,^20^, ^21^ this was the first confirmation study to evaluate the efficacy and safety of brexpiprazole in Japanese patients.
2. **Interpretations**: Not only brexpiprazole 2 mg/day but also 1 mg/day demonstrated statistically significant improvement (the latter was not tested or had no statistical significance in the global studies). Both doses of brexpiprazole were generally well tolerated, although the incidence of adverse events was higher in our study than in the global studies, which might be due to the different patient characteristics.
3. **Future directions**: The long‐term extended study (24 weeks in total) will continue to evaluate the safety and efficacy of brexpiprazole.

## 2 METHODS

### 2.1 Patients

For the key inclusion criteria, eligible patients were 55‐ to 90‐years‐old, had a diagnosis of Alzheimer's type dementia according to the Diagnostic and Statistical Manual of Mental Disorders, Fifth Edition (DSM‐5),^22^ a diagnosis of probable Alzheimer's disease by the National Institute of Neurological and Communicative Disorders and Stroke–Alzheimer's Disease and Related Disorders Association,^23^ and a caregiver who could collect patient's information required for evaluations. Moreover, patients must have had a total score of 1–22 on the Mini‐Mental State Examination (MMSE)^24^ at the screening test, a score of ≥4 in agitation/aggression domain score (frequency × severity) of the Neuropsychiatric Inventory—Nursing Home version (NPI‐NH)^25^ or NPI^26^ at the screening test and the baseline assessment, and continuous or frequent recurrence of agitation affecting daily life for ≥2 weeks before both the screening test and the baseline assessment (the definition of agitation was in accordance with the International Psychogeriatric Association's consensus definition^2^). In addition, patients were required to have three or more occasions of verbal aggression or physical aggression during the 2 weeks before the baseline assessment. For the key exclusion criteria, patients who had dementia other than Alzheimer's type or memory impairment were excluded. Patients diagnosed with delirium, schizophrenia spectrum, other psychotic disorders, bipolar disorder, bipolar‐related disorders, and major depressive disorder, according to DSM‐5, were also excluded. Moreover, patients who had previously received antipsychotic medication and whose condition had been judged to be treatment resistant were not allowed to participate in this study.

This study was registered with ClinicalTrials.gov (identifier: NCT03620981) and conducted in compliance with the International Council for Harmonisation Good Clinical Practice, the Declaration of Helsinki, and local regulations. The protocol, its amendments, and informed consent forms were reviewed and approved by each site's institutional review boards (for the names of the institutional review boards, see Supporting Information). Written informed consent was obtained from patients and/or their legal representatives and caregivers after the procedures had been fully explained. The study was conducted at 120 sites nationwide in Japan, including not only large hospitals but also small clinics, with diversity, equity, and inclusion in consideration.

### 2.2 Study design

This was a phase 2/3, multicenter, randomized, double‐blind, placebo‐controlled, parallel‐group comparison study. The study comprised a screening period of up to 42 days, a treatment period of 10 weeks, and a follow‐up period of 28 days. Eligible patients were randomized in a 3:4:4 ratio to brexpiprazole 1 or 2 mg, or placebo for 10 weeks. Brexpiprazole was initiated at 0.5 mg in Week 1, increased to 1 mg in Week 2 (Day 8), and further increased to 2 mg in Week 3 (Day 15) for the 2 mg group. Brexpiprazole or placebo was taken orally once daily (Figure S1). The 10‐week treatment period was determined on the basis of preceding studies, where it took 8 weeks to confirm the sufficient efficacy of brexpiprazole after 2‐week or 4‐week titration to reach the dose of 2 mg/day.^20^, ^21^ In alignment with the preceding study,^21^ our study adopted 2‐week titration to reach the dose of 2 mg/day and an additional 8 weeks for treatment (in total 10‐week treatment period). Concomitant medications of antidementia drugs, narcotic analgesics, beta‐blockers, and sleeping drugs were allowed with restrictions (Table S1).

Dynamic allocation was applied according to the following background factors: medical care category (hospital or outpatient [care facility or home]), with or without antipsychotics as prior medication (definition was drugs used in 6 months before the baseline assessment), and <56 or ≥56 CMAI total score at the baseline assessment. The randomization code was generated by a computer, and patients were assigned accordingly. Patients, caregivers, investigators, and sponsor personnel, including those involved in data analysis, were blinded to treatment assignments.

### 2.3 Assessments

#### 2.3.1 Primary endpoint

The primary endpoint was the change in CMAI total score from baseline to Week 10.^27^, ^28^, ^29^ CMAI total score was calculated as the sum of the 29 agitated behavior items, with each item scored from 1 (never) to 7 (a few times an hour), giving a total score of 29 to 203 points.

#### 2.3.2 Secondary endpoints

The secondary endpoints were changed in CMAI subscale (aggressive behavior, physically nonaggressive behavior, and verbally agitated behavior) scores from baseline to Week 10, change in Clinical Global Impression—Severity of Illness (CGI‐S) score from baseline to Week 10, and Clinical Global Impression—Global Improvement (CGI‐I) score at Week 10.^30^

#### 2.3.3 Other endpoints

The other endpoints were Alzheimer's Disease Cooperative Study—Activities of Daily Living (ADCS‐ADL)^31^ and MMSE.

#### 2.3.4 Safety endpoints

The safety endpoints were treatment‐emergent adverse events (TEAEs), physical examination, laboratory tests, vital signs, body weight, 12‐lead electrocardiography, pregnancy test (only patients with childbearing potential), Drug‐Induced Extra‐Pyramidal Symptoms Scale (DIEPSS),^32^ Abnormal Involuntary Movement Scale (AIMS),^30^ Barnes Akathisia Rating Scale (BARS),^33^ and Sheehan Suicidality Tracking Scale (S‐STS).^34^

### 2.4 Statistical analysis

Sample size calculations were based on an assumption of difference for brexpiprazole 2 mg versus placebo of −5.35 (standard deviation [SD] = 15.06) in change in CMAI total score from baseline to Week 10. Sample sizes of 111, 148, and 148 evaluable patients, each in brexpiprazole 1 or 2 mg, or placebo groups, respectively (3:4:4 ratio), was expected to yield ≥86.1% power for brexpiprazole 2 mg versus placebo and ≥80.5% power for brexpiprazole 1 mg versus placebo, at a two‐sided alpha of 0.05.

The full analysis set consisted of patients who were randomized, administered at least one dose of study treatment, and had a CMAI total score at baseline and at least one occasion after baseline. The primary analysis of the primary endpoint was conducted by mixed models for repeated measures (MMRM), based on the assumption of missing at random, using an observed cases data set that did not impute missing values. The model included treatment group, visit, medical care category, prior use of antipsychotics, and interaction between treatment group and visit as factors, baseline, and interaction between baseline and visit as covariates with an unstructured covariance structure. In this MMRM analysis, least squares (LS) mean of each group and LS mean difference and its two‐sided 95% confidence interval (CI) of brexpiprazole 1 or 2 mg versus placebo were calculated. To control the overall type I error, multiplicity was adjusted by a hierarchical testing procedure, in which the comparison of brexpiprazole 2 mg versus placebo was tested at a significance level of 0.05 (two‐sided) first, and only if the *p*‐value was less than 0.05, the comparison of brexpiprazole 1 mg versus placebo was tested at the significance level 0.05. The secondary endpoints, change in CMAI subscale scores, and change in CGI‐S score, each from baseline to Week 10, were analyzed similarly to the primary endpoint analysis. CGI‐I score at Week 10 was analyzed, using the last observation carried forward dataset, by Cochran Mantel Haenszel row mean scores test, stratified by medical care category and with or without antipsychotics as prior medication. The safety analysis set consisted of patients who were randomized and administered at least one dose of study treatment. The incidences of TEAEs in each group and the total brexpiprazole group (1 and 2 mg combined) were summarized. All analyses were performed using SAS software version 9.4 (SAS Institute, Cary, NC, USA).[Fig alz14282-fig-0001]

## 3 RESULTS

### 3.1 Patients

**FIGURE 1 alz14282-fig-0001:**
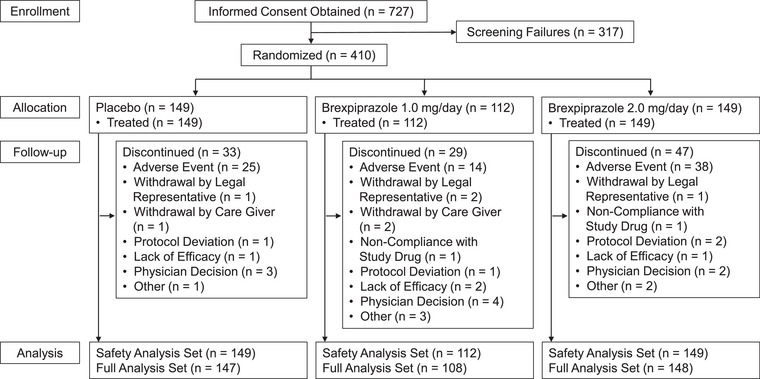
Patient disposition. The safety analysis set consisted of patients who were randomized and administered at least one dose of study medication. The full analysis set consisted of patients who were randomized, administered at least one dose of study medication, and had a CMAI total score at baseline and at least one occasion after baseline. CMAI, Cohen‐Mansfield Agitation Inventory.

This study was conducted from August 20, 2018, to May 15, 2023. A total of 727 patients provided informed consent. Of these, 410 patients were eligible and randomized: 112, 149, and 149 patients to brexpiprazole 1 or 2 mg, or placebo, respectively (hereafter, treatment groups are described in this order). All randomized patients received study medication at least once, and the study completion rates were 74.1% (83/112), 68.5% (102/149), and 77.9% (116/149). In all groups, the most common reason for study discontinuation was adverse events (AEs): 12.5% (14/112), 25.5% (38/149), and 16.8% (25/149). All randomized patients were analyzed for safety, and 108, 148, and 147 patients in each treatment group were analyzed for efficacy (Figure 1).

The baseline demographic and clinical characteristics were similar across treatment groups (Table 1). The mean CMAI total scores were 62.1, 64.1, and 62.7, and the mean CGI‐S scores were 4.5, 4.6, and 4.6.

**TABLE 1:** Demographic and baseline clinical characteristics (FAS).

Characteristics	Placebo (*N* = 147)		Brex 1 mg (*N* = 108)		Brex 2 mg (*N* = 148)		Total Brex (*N* = 256)		Total (*N* = 403)	
Demographic										
Age (years), mean (SD)	80.5	(6.5)	79.3	(7.4)	80.2	(6.4)	79.8	(6.8)	80.1	(6.7)
Sex										
Male, *n* (%)	62	(42.2)	41	(38.0)	43	(29.1)	84	(32.8)	146	(36.2)
Female, *n* (%)	85	(57.8)	67	(62.0)	105	(70.9)	172	(67.2)	257	(63.8)
Weight (kg), mean (SD)	47.82	(8.60)	47.88	(9.78)	48.32	(9.45)	48.13	(9.58)	48.02	(9.22)
BMI (kg/m^2^), mean (SD)	20.59	(2.80)	20.65	(3.28)	21.05	(3.53)	20.88	(3.42)	20.77	(3.21)
Clinical										
Duration										
Alzheimer's disease (months)^a^, mean (SD)	61.2	(39.9)	65.6	(45.1)	66.0	(40.0)	65.8	(42.1)	64.1	(41.3)
Agitation from Alzheimer's disease (months)^a^, mean (SD)	25.7	(27.2)	22.7	(23.2)	25.1	(25.2)	24.1	(24.4)	24.7	(25.4)
NPI/NPI‐NH agitation/aggression score, mean (SD)	7.6	(2.5)	7.3	(2.6)	7.6	(2.6)	7.5	(2.6)	7.5	(2.6)
CGI‐S score, mean (SD)	4.6	(1.0)	4.5	(0.8)	4.6	(0.9)	4.6	(0.9)	4.6	(0.9)
CMAI total score, mean (SD)	62.7	(11.7)	62.1	(11.3)	64.1	(12.9)	63.3	(12.3)	63.1	(12.0)
MMSE total score, mean (SD)	10.6	(6.3)	11.7	(6.5)	11.5	(6.3)	11.6	(6.4)	11.2	(6.3)
Medical care category										
Hospital, *n* (%)	102	(69.4)	75	(69.4)	102	(68.9)	177	(69.1)	279	(69.2)
Care facility, *n* (%)	12	(8.2)	7	(6.5)	11	(7.4)	18	(7.0)	30	(7.4)
Home, *n* (%)	33	(22.4)	26	(24.1)	35	(23.6)	61	(23.8)	94	(23.3)
Prior medications (antipsychotic)	98	(66.7)	71	(65.7)	97	(65.5)	168	(65.6)	266	(66.0)

*Note*: Percentages are based on the number of patients in the treatment group.

Abbreviations: BMI, body mass index; Brex, brexpiprazole; CGI‐S, Clinical Global Impression—Severity of Illness; CMAI, Cohen‐Mansfield Agitation Inventory; FAS, full analysis set; MMSE, Mini‐Mental State Examination; NPI, Neuropsychiatric Inventory; NPI‐NH, Neuropsychiatric Inventory—Nursing Home version; SD, standard deviation.

^a^ Months derived based on (date of assessment—estimated date of onset + 1)/30. Any unknown month or day of onset is imputed with June or 15, respectively.

### 3.2 Primary endpoint

**FIGURE 2:**
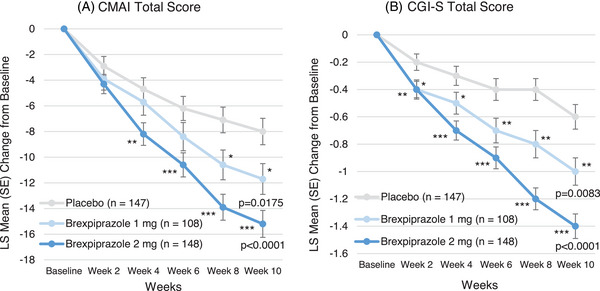
Changes in CMAI and CGI‐S total scores from baseline to Week 10 (FAS). (A) MMRM analysis. The mean (SD) CMAI total scores at baseline were 62.1 (11.3) for brexpiprazole 1 mg, 64.1 (12.9) for brexpiprazole 2 mg, and 62.7 (11.7) for placebo. (B) MMRM analysis. The mean (SD) CGI‐S total scores at baseline were 4.5 (0.8) for brexpiprazole 1 mg, 4.6 (0.9) for brexpiprazole 2 mg, and 4.6 (1.0) for placebo. The scale of CGI‐S is 1 = normal, not at all ill, 2 = borderline mentally ill, 3 = mildly ill, 4 = moderately ill, 5 = markedly ill, 6 = severely ill, and 7 = among the most extremely ill patients. *p‐value < 0.05, **p‐value < 0.01, ***p‐value < 0.001. CGI‐S, Clinical Global Impression—Severity of Illness; CMAI, Cohen‐Mansfield Agitation Inventory; FAS, full analysis set; LS, least squares; MMRM, mixed models for repeated measures; SD, standard deviation; SE, standard error.

For the primary efficacy endpoint of change in CMAI total score from baseline to Week 10, the LS mean changes (standard error [SE]) in the brexpiprazole 1 mg, 2 mg, and placebo groups were −11.7 (1.20), −15.2 (1.05), and −8.0 (1.03), respectively. Both brexpiprazole groups demonstrated statistically significant improvement versus the placebo group (2 mg: LS mean difference −7.2 [95% CI: −10.0, −4.3], *p*‐value < 0.0001, 1 mg: LS mean difference −3.7 [95% CI: −6.8, −0.7], *p*‐value = 0.0175). The treatment difference versus placebo was greater in brexpiprazole 2 mg than in 1 mg (Figure 2). By timepoint, both brexpiprazole groups showed decreases of LS mean changes from Week 2, and in the brexpiprazole 2 mg group, improvement was observed early at Week 4 (*p*‐value < 0.01) and continued at Week 6 (*p*‐value < 0.001), Week 8 (*p*‐value < 0.0001), and Week 10 (*p*‐value < 0.0001), whereas in the brexpiprazole 1 mg group, such improvement was observed later at Week 8 and Week 10 (*p*‐value < 0.05) (Figure 2).

### 3.3 Secondary endpoints

**FIGURE 3:**
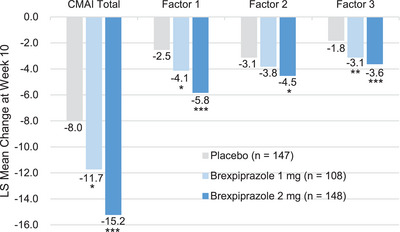
Change in CMAI subscale scores from baseline to Week 10 (FAS). MMRM analysis. Factor 1: aggressive behavior (both physical and verbal). Factor 2: physically nonaggressive behavior (excessive motor activity). Factor 3: verbally agitated behavior. *p‐value < 0.05, **p‐value < 0.01, ***p‐value < 0.001. CMAI, Cohen‐Mansfield Agitation Inventory; FAS, full analysis set; LS, least squares; MMRM, mixed models for repeated measures.

The secondary efficacy endpoint of change in CMAI subscale scores from baseline to Week 10 showed similar results. In all subscale scores of aggressive behavior, physically nonaggressive behavior, and verbally agitated behavior, improvements were observed in the brexpiprazole 1mg and 2 mg groups versus the placebo group (*p*‐value < 0.05 each), except the brexpiprazole 1 mg group in physically nonaggressive behavior score (numerically improved [*p*‐value > 0.05]). The treatment difference versus placebo was greater in brexpiprazole 2 mg than in 1 mg (Figure 3).

The other secondary efficacy endpoints of change in CGI‐S score from baseline to Week 10 and CGI‐I score at Week 10 showed similar results. For both endpoints, improvements were observed in the brexpiprazole 1 mg and 2 mg groups versus the placebo group (*p*‐value < 0.01 and *p*‐value < 0.0001, respectively); and the treatment difference versus placebo was greater in brexpiprazole 2 mg than 1 mg (Figure 2 and Figure S2).

### 3.4 Other endpoints

Regarding ADCS‐ADL and MMSE, the LS mean changes (SE) of the total score from baseline to last visit were small and no clinically meaningful changes were observed: in the order of the brexpiprazole 1 mg, 2 mg, and placebo groups, −0.6 (0.52), −0.8 (0.46), and 0.7 (0.44) for ADCS‐ADL, and −0.7 (0.34), −0.3 (0.30), and 0.0 (0.29) for MMSE, respectively.

### 3.5 Safety endpoints

The incidences of TEAEs in the brexpiprazole 1 mg, 2 mg, and placebo groups were 76.8% (86/112), 84.6% (126/149), and 73.8% (110/149), respectively. Two TEAEs with an outcome of death occurred in the brexpiprazole 1 mg group, whereas no deaths were observed in the brexpiprazole 2 mg and placebo groups. The resulting overall incidence of deaths in the brexpiprazole group was 0.8% (2/261). The incidences of serious TEAEs in each group were 6.3% (7/112), 6.0% (9/149), and 4.7% (7/149); those of severe TEAEs were 7.1% (8/112), 10.1% (15/149), and 6.7% (10/149); and those of TEAEs leading to discontinuation of study treatment were 12.5% (14/112), 25.5% (38/149), and 16.8% (25/149), respectively (Table 2).

**TABLE 2:** Summary of TEAEs (safety analysis set).

	Placebo (*N* = 149)		Brex 1 mg (*N* = 112)		Brex 2 mg (*N* = 149)		Total Brex (*N* = 261)	
	*n*	(%)^a^	*n*	(%)^a^	*n*	(%)^a^	*n*	(%)^a^
Patients with TEAEs^b^	110	(73.8)	86	(76.8)	126	(84.6)	212	(81.2)
Patients with TEAEs with an outcome of death	0	(0.0)	2	(1.8)	0	(0.0)	2	(0.8)
Patients with serious TEAEs	7	(4.7)	7	(6.3)	9	(6.0)	16	(6.1)
Patients with severe TEAEs	10	(6.7)	8	(7.1)	15	(10.1)	23	(8.8)
Patients with discontinuation of study treatment due to TEAEs	25	(16.8)	14	(12.5)	38	(25.5)	52	(19.9)
TEAEs with an incidence ≥5% in the brexpiprazole 1 mg or 2 mg group (preferred term)								
Somnolence	3	(2.0)	9	(8.0)	24	(16.1)	33	(12.6)
Bradykinesia	0	(0.0)	8	(7.1)	20	(13.4)	28	(10.7)
Insomnia	22	(14.8)	16	(14.3)	18	(12.1)	34	(13.0)
Salivary hypersecretion	1	(0.7)	1	(0.9)	18	(12.1)	19	(7.3)
Muscle rigidity	1	(0.7)	9	(8.0)	16	(10.7)	25	(9.6)
Gait disturbance	1	(0.7)	5	(4.5)	15	(10.1)	20	(7.7)
Fall	9	(6.0)	9	(8.0)	14	(9.4)	23	(8.8)
Pyrexia	8	(5.4)	9	(8.0)	11	(7.4)	20	(7.7)
Sedation complication	2	(1.3)	3	(2.7)	11	(7.4)	14	(5.4)
Contusion	19	(12.8)	9	(8.0)	9	(6.0)	18	(6.9)
Decreased appetite	1	(0.7)	7	(6.3)	8	(5.4)	15	(5.7)
Skin abrasion	9	(6.0)	4	(3.6)	8	(5.4)	12	(4.6)
Back pain	3	(2.0)	6	(5.4)	6	(4.0)	12	(4.6)
Dystonia	1	(0.7)	7	(6.3)	4	(2.7)	11	(4.2)

*Note*: Medical Dictionary for Regulatory Activities v25.0 was used.

Abbreviations: Brex, brexpiprazole; TEAEs, treatment‐emergent adverse events.

^a^ Percentages are based on the number of patients treated with double‐blind study medication.

^b^ All adverse events that started after start of the double‐blind study drug treatment. Adverse events are counted once, per term, for the most severe of multiple occurrences of a specific Medical Dictionary for Regulatory Activities preferred term.

TEAEs with an incidence of ≥5% in the total brexpiprazole group were somnolence, bradykinesia, insomnia, salivary hypersecretion, muscle rigidity, gait disturbance, fall, pyrexia, sedation complication, contusion, and decreased appetite. The severity of most TEAEs was mild or moderate, and no new safety signals were observed (Table 2).

The two TEAEs that resulted in death were cardiac death and pneumonia aspiration, and both were considered not related to brexpiprazole 1 mg by the investigators. The only serious TEAE that occurred in two or more patients in either brexpiprazole group was pneumonia aspiration (1.8% [2/112] in brexpiprazole 1 mg, 1.3% [2/149] in brexpiprazole 2 mg), of which only one event in the brexpiprazole 1 mg group was considered related to brexpiprazole by the investigator. As an AE of interest, the incidences of extrapyramidal symptom (EPS)–related TEAEs in the brexpiprazole 1 mg, 2 mg, and placebo groups were 25.9%, 32.2%, and 7.4%, respectively. The LS mean changes (SE) of DIEPSS total score from baseline to last visit were 1.0 (0.22), 1.6 (0.19), and 0.0 (0.19). Those of AIMS scores and BARS “Global Clinical Assessment of Akathisia” scores were < 1.0 in all groups. S‐STS, another AE of interest, had no clinically meaningful change.

Regarding laboratory values, vital signs, body weight, body mass index (BMI), and 12‐lead electrocardiography, no clinically meaningful changes and no remarkable differences were observed in all groups. The proportion of patients with a statistical analysis plan–defined clinically important increase/decrease ≥7% was analyzed: in the brexpiprazole 1 mg, 2 mg, and placebo groups, weight increase was observed in 2.9% (3/104), 6.2% (9/145), and 2.8% (4/145), whereas weight decrease was observed in 4.8% (5/104), 4.1% (6/145), and 2.8% (4/145), respectively.

## 4 DISCUSSION

As of 2023, brexpiprazole was the first and only FDA‐approved drug for the treatment of agitation associated with dementia due to Alzheimer's disease, and in 2024, brexpiprazole was approved for the same indication in Canada and the Philippines. However, brexpiprazole is not yet approved in other countries, and clinical knowledge of brexpiprazole globally is still evolving. This study was the first confirmation study in Japanese patients with AAD to evaluate the efficacy, safety, and tolerability of brexpiprazole. On the primary efficacy endpoint (change in CMAI total score from baseline to Week 10), both brexpiprazole 1 mg and 2 mg demonstrated statistically significant improvement versus placebo, and the treatment effect was greater in brexpiprazole 2 mg than 1 mg. On the secondary efficacy endpoints (change in CMAI subscale scores and change in CGI‐S score, each from baseline to Week 10, and CGI‐I score at Week 10), similar results were observed, except that the improvement of the CMAI subscale score factor 2 (physically nonaggressive behavior) in the brexpiprazole 1 mg group was numerical. It is notable that in CMAI subscale scores, not only aggressive behavior and verbally agitated behavior but also physically nonaggressive behavior was improved. In addition, improvements in LS mean changes of CMAI total score were observed earlier in brexpiprazole 2 mg (from Week 4, *p*‐value < 0.01) than in 1 mg (from Week 8, *p*‐value < 0.05). These results indicate that both brexpiprazole 1 mg and 2 mg are efficacious and that treatment effect can be expected earlier with increased dose. The incidence of TEAEs was greater in brexpiprazole 2 mg (84.6%) than in 1 mg (76.8%) and placebo (73.8%), and the incidence of TEAEs leading to discontinuation of study treatment was greater in 2 mg (25.5%) than in 1 mg (12.5%) and placebo (16.8%). Most TEAEs were mild to moderate in severity and both doses of brexpiprazole were generally well tolerated.

In a similar preceding study in AAD patients, the overall brexpiprazole group with a fixed dose of 2 or 3 mg demonstrated statistically significant improvement versus the placebo group in the primary efficacy endpoint (change in CMAI total score from baseline to Week 12). In the overall brexpiprazole group and the placebo group, the incidence of TEAEs was 40.7% and 31.0%, respectively, and the incidence of TEAEs leading to discontinuation of study treatment was 5.3% and 4.3%, respectively (ClinicalTrials.gov Identifier: NCT03548584).^21^ It is notable that in the preceding study ≥90% of patients were White, mean age was ≈73–75 years of age, and mean body weight was ≈70–71 kg, whereas in our study all patients were Asian (Japanese), mean age was higher (≈80 years of age), and mean body weight was lower (≈48 kg) than the preceding study. The higher incidence of TEAEs in both the brexpiprazole groups and the placebo group in our study may be attributable to the differences in these patient population characteristics. In a preceding study with a fixed dose of 1 or 2 mg, brexpiprazole 2 mg demonstrated statistically significant improvement in the similar primary efficacy endpoint (change in CMAI total score from baseline to Week 12), but brexpiprazole 1 mg did not (ClinicalTrials.gov Identifier: NCT01862640).^20^ In our study, both brexpiprazole 1 mg and 2 mg demonstrated statistically significant improvement.

As for the safety of atypical antipsychotics in this patient population, risk of death, EPS, weight gain, and metabolic syndrome have been investigated.^35^, ^36^ Two deaths during this study (cardiac death and pneumonia aspiration) were considered not related to brexpiprazole by the investigators, and in terms of suicidality, S‐STS had no clinically meaningful change. Brexpiprazole had a higher incidence of EPS‐related TEAEs versus placebo. However, from the viewpoint of EPS rating scales, such as DIEPSS, AIMS, and BARS, little or no impact was observed by brexpiprazole. Body weight and BMI had no clinically meaningful changes.

Cerebrovascular AEs, somnolence/sedation, falls/fracture/injury, and urinary incontinence/urinary tract infection are also known AEs of atypical antipsychotics.^35^ In this study, TEAEs of somnolence and sedation complication had an incidence of ≥5% and ≥2 times the rate of placebo in either brexpiprazole group (somnolence [1 mg: 8.0%, 2 mg: 16.1%, placebo: 2.0%], sedation complication [1 mg: 2.7%, 2 mg: 7.4%, placebo: 1.3%]), but others did not show a higher incidence in brexpiprazole versus placebo.

Although we cannot compare directly due to the different study designs, the mortality risk did not increase for 10 weeks regardless of antipsychotic drug in the large‐scale prospective cohort study,^37^ to which our study showed a similar tendency.

In this study, the percentage of patients in a care facility or at home was ≈30% in total. If the severity and frequency of agitation can be controlled by brexpiprazole with outpatient care, early hospitalization may be avoided.

This study included patients with mild, moderate, and severe cognitive dysfunction by the inclusion criterion for MMSE (patients must have had a total score of 1–22) in all medical care categories (hospital, care facility, and home), supporting the generalizability of the results. In terms of age, sex, and body weight, no distinctive differences were observed in the patient characteristics between our study and the large‐scale prospective cohort study with ≈10,000 patients.^37^

Considering that the efficacy results demonstrated a statistically significant treatment effect and that the safety results showed no new safety signals, brexpiprazole appears to have a favorable benefit/risk profile for the treatment of AAD in Japanese patients.

The limitations of this study are as follows. CMAI, NPI, NPI‐NH, and ADCS‐ADL were completed by investigators based on information from caregivers (to minimize the influences caused by different caregivers, the protocol defined requirements for setting a primary caregiver, such as being available at least 4 days/week and 4 h/day for patient observation, etc.). Patients with certain concurrent diseases and concomitant medications were excluded, thereby limiting the generalizability of the results. This study was conducted in Japanese patients, and thus caution is necessary when extrapolating the results to other racial/ethnic groups. The persistent blocking of dopamine D_2_ receptors results in dopamine hypersensitivity, which is considered one of the causes of tardive dyskinesia,^38^ and brexpiprazole is a partial agonist at dopamine D_2_ receptors, which means brexpiprazole also has an antagonistic function against dopamine D_2_ receptors. In this study, no tardive dyskinesia was reported in any of the treatment groups, but the treatment period was 10 weeks. To address this point and to investigate the long‐term (24 weeks) efficacy and safety of brexpiprazole in Japanese patients with AAD, an extension study was conducted. The results will be published separately.

In conclusion, the treatment of brexpiprazole 2 mg/day for 10 weeks demonstrated statistically significant improvement in the primary efficacy endpoint (mean change in CMAI total score) versus placebo. The brexpiprazole 1 mg/day did the same, and the outcomes of the secondary efficacy endpoints were also consistent with those of the primary efficacy endpoint. Treatment with brexpiprazole 1 mg/day and 2 mg/day for 10 weeks was generally well tolerated. These results showed that brexpiprazole treatment, starting from 0.5 mg/day and increasing to 1 mg/day and 2 mg/day, was efficacious without remarkable safety concerns.

## CONFLICT OF INTEREST STATEMENT

Yu Nakamura has received speakers’ honoraria, manuscript fee, research support, or scholarship donation from Otsuka Pharmaceutical Co., Ltd., Meiji Seika Pharma Co., Ltd., Viatris Pharmaceutical K.K., Eisai Co., Ltd., Takeda Pharmaceutical Co., Ltd., Teikoku Pharmaceutical K.K., Kowa Company Ltd., Mochida Pharmaceutical Co., Ltd., Towa Pharmaceutical Co., Ltd, MSD K.K., Biogen Japan Ltd., and Daiichi Sankyo Company Ltd. Jun Adachi, Naoki Hirota, Katsuhiro Iba, Koichi Shimizu, Masami Nakai, Kaneyoshi Takahashi, and Naoki Mori are full‐time employees of Otsuka Pharmaceutical Co., Ltd. Author disclosures are available in the Supporting Information.

## CONSENT STATEMENT

All patients provided written informed consent.

## Supporting information

Supporting Information

Supporting Information

Supporting Information

Supporting Information

Supporting Information
