# Supplementary material for: Brexpiprazole treatment for agitation in Alzheimer's dementia: A randomized study
Source: Alzheimers Dement. 2024 Oct 6;20(11):8002–11. doi: 10.1002/alz.14282 (PMC11567808; doi:10.1002/alz.14282)
Supplement: Supplementary file 1 — Supporting Information [file ALZ-20-8002-s005.pdf]

Supplemental Figure 1. Study Design

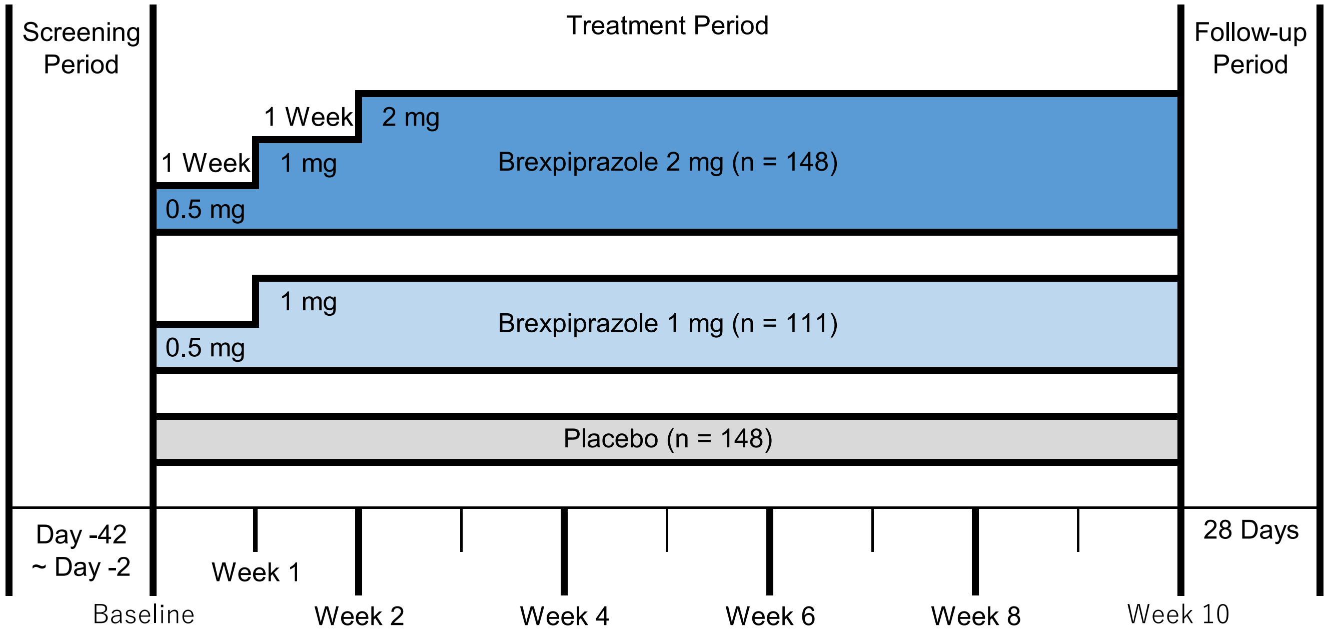

NOTE. Eligible patients were randomized in a 3:4:4 ratio to brexpiprazole 1 mg, 2 mg, or placebo for 10 weeks. Brexpiprazole was initiated at 0.5 mg in Week 1, increased to 1 mg in Week 2 (Day 8), and further increased to 2 mg in Week 3 (Day 15) for the 2 mg group. Brexpiprazole or placebo was taken orally once daily, preferably at the same time each day, regardless of meals. Visits occurred at Week 1 and every 2 weeks afterwards. Antidementia drugs were permitted during the study, as long as the dose was stable for ≥3 months prior to the baseline assessment and was not changed until the assessments at Week 10 or early termination visit were completed.
