# Supplementary material for: Brexpiprazole treatment for agitation in Alzheimer's dementia: A randomized study
Source: Alzheimers Dement. 2024 Oct 6;20(11):8002–11. doi: 10.1002/alz.14282 (PMC11567808; doi:10.1002/alz.14282)
Supplement: Supplementary file 2 — Supporting Information [file ALZ-20-8002-s001.pdf]

Supplemental Figure 2. CGI-I Score at Week 10 (FAS)

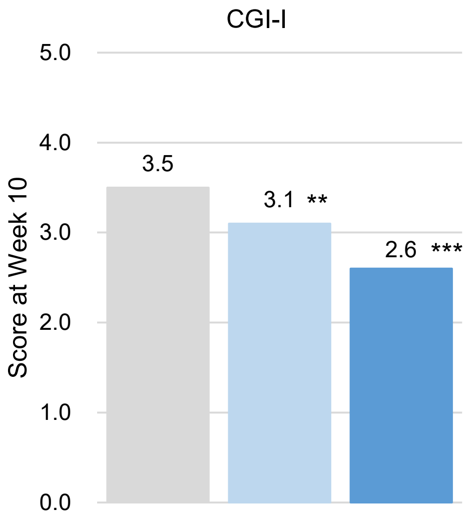

	Placebo (n = 147)	Brex 1 mg (n = 108)	Brex 2 mg (n = 148)
Mean at Week 10 (SD)	3.5 (1.4)	3.1 (1.3)	2.6 (1.2)
Treatment Difference		-0.5	-0.9
(95% CI)		(-0.8, -0.1)	(-1.2, -0.6)
P-value		0.0052	<0.0001

NOTE. Cochran Mantel Haenszel row mean scores test. The scale of CGI-I is 1=very much improved, 2=much improved, 3=minimally improved, 4=no change, 5=minimally worse, 6=much worse, 7=very much worse. Brex: brexpiprazole, CGI-I: Clinical Global Impression - Global Improvement, CI: confidence interval, FAS: full analysis set, SD: standard deviation. **p<0.01, ***p<0.001.
