# Supplementary material for: Brexpiprazole treatment for agitation in Alzheimer's dementia: A randomized study
Source: Alzheimers Dement. 2024 Oct 6;20(11):8002–11. doi: 10.1002/alz.14282 (PMC11567808; doi:10.1002/alz.14282)
Supplement: Supplementary file 3 — Supporting Information [file ALZ-20-8002-s004.docx]

Supplemental Table 1. Concomitant Medications Allowed with Restrictions

| **Types of Drugs** | **Restrictions** |
| --- | --- |
| Antidementia drugs | Antidementia drugs were permitted during the study, as long as the dose was stable for ≥3 months prior to the baseline assessment and was not changed until the assessments at Week 10 or early termination visit were completed. |
| Narcotic analgesics | Narcotic analgesics were permitted from the baseline assessments until the assessments at Week 10 or early termination visit were completed, as long as it was not for the treatment of agitation (e.g. pain management, tooth extraction, etc.). |
| Beta blockers | - Treatment for concurrent diseases other than mental disorders (e.g. cardiovascular diseases, etc.)   The use was permitted during the study, as long as it was used before informed consent was provided, the dose was stable for ≥30 days prior to the baseline assessment, and the type/dose was not changed until the assessments at Week 10 or early termination visit were completed (however, the discontinuation of use or the decrease of dose was permitted, if necessary due to the occurrence of TEAEs by the beta blockers, or due to the complete resolution of symptoms.).   - Treatment for extrapyramidal symptoms-related TEAE   If medication was needed, up to 60 mg/day as propranolol was permitted. However, the use was prohibited within 12 hours before the efficacy and safety evaluations. |
| Sleeping drugs | - Ultra-short-acting non-benzodiazepine sleeping drugs (zolpidem, zopiclone, eszopiclone), and ramelteon   The use was permitted during the study, as long as the dose was stable for ≥30 days prior to the baseline assessment, and the type/dose was not changed until the assessments at Week 10 or early termination visit were completed (however, the discontinuation of use or the decrease of dose was permitted, if necessary due to the occurrence of TEAEs by the sleeping drugs, or due to the complete resolution of insomnia symptoms.). However, the use was prohibited within 8 hours before the efficacy and safety evaluations.   - Treatment for insomnia symptoms-related TEAE   If medication was needed, one of the ultra-short-acting non-benzodiazepine sleeping drugs (zolpidem, zopiclone, eszopiclone) was permitted (change of which to use was allowed). However, the use was prohibited within 8 hours before the efficacy and safety evaluations. |

Abbreviations: TEAE, treatment-emergent adverse event.
