# Supplementary material for: Brexpiprazole treatment for agitation in Alzheimer's dementia: A randomized study
Source: Alzheimers Dement. 2024 Oct 6;20(11):8002–11. doi: 10.1002/alz.14282 (PMC11567808; doi:10.1002/alz.14282)
Supplement: Supplementary file 5 — Supporting Information [file ALZ-20-8002-s003.pdf]

List of IECs or IRBs

Trial Site Number	IEC/IRB Name	IEC/IRB Chair Name
Local IRB – Japan Site 004	NHO Kanazawa Medical Center IRB<br>1-1, Shimoishibiki-machi, Kanazawa-shi, Ishikawa 920-8650, Japan	Ichiro Onishi
Local IRB – Japan Site 006	Hospital of the University of Occupational and Environmental Health, Japan IRB<br>1-1, Iseigaoka, Yahatanishi-ku, Kitakyushu-shi, Fukuoka 807-8556, Japan	Koichi Kusuhara
Local IRB – Japan Site 011	IRB of Yuge Hospital<br>5-12-25 ,Yuge, Kita-ku, Kumamoto-shi, Kumamoto 861-8002, Japan	Sachi Yamashiro
Local IRB – Japan Site 014	National Hospital Organization Ryukyu Hospital IRB<br>7958-1, Kin, Kunigami-gun, Kin-cho, Okinawa 904-1201, Japan	Yohachiro Ohama
Local IRB – Japan Site 015	Teine Keijinkai Hospital IRB<br>1-12-1-40, Maeda, Teine-ku, Sapporo-shi, Hokkaido 006-8555, Japan	Satoshi Yuda
Local IRB – Japan Site 016	Akita City Hospital IRB<br>4-30, Kawamoto Matsuoka-machi, Akita-shi, Akita 010-0933, Japan	Toshiya Ishida
Local IRB – Japan Site 020	Kohnodai Hospital National Center for Global Health and Medicine IRB<br>1-7-1, Konodai, Ichikawa-shi, Chiba 272-8516, Japan	Hidekatsu Yanai
Local IRB – Japan Site 021	Sankeikai Nishigahara Hospital lRB<br>2-46-9, Nishigahara, Kita-Ku, Tokyo 114-0024, Japan	Masahiro Shigeta
Central – Japan Site 024	Suzuki Internal & Circulatory Medical Clinic IRB<br>1-39-5, Sangenjaya, Setagaya-ku, Tokyo 154-0024, Japan	Satoru Tani
Central – Japan Site 028	Medical Corporation Tachikawa Medical Center Tachikawa General Hospital IRB<br>1-24, Asahioka, Nagaoka-shi, Niigata 940-8621, Japan	Tetsuya Tada
Central IRB – Japan Site 029	Fukui General Hospital IRB<br>58-16-1, Egami-cho, Fukui-shi, Fukui 910-8561, Japan	Tetsuro Tsuji
Local IRB – Japan Site 032	Osaka Medical and Pharmaceutical University Hospital IRB<br>2-7, Daigakumachi, Takatsuki-shi, Osaka 569-8686, Japan	Ryuichi Saura
Local IRB – Japan Site 034	Kansai Medical University Medical Center IRB<br>10-15, Fumizono-cho, Moriguchi-shi, Osaka 570-8507, Japan	Toshihito Seki
Local IRB – Japan Site 035	National Hospital Organization Tottori Medical Center IRB<br>876, Mitsu, Tottori-shi, Tottori 689-0203, Japan	Shinjiro Akaboshi
Local IRB – Japan Site 037	Koseikai Kusatsu Hospital IRB	Shuji Miyatake

Until the information herein is released by Otsuka to the public domain, the contents of this document are Otsuka confidential information and should not be duplicated or re-distributed without prior written consent of Otsuka.

Trial Site Number	IEC/IRB Name	IEC/IRB Chair Name
	10-1, Kusatsu Umegadai, Nishi-ku, Hiroshima-shi, Hiroshima 733-0864, Japan	
Local IRB – Japan Site 038	Hayakawa Clinic IRB 2-7-4, Hiroobiro, Kure-shi, Hiroshima 737-0111, Japan	Shigeto Yamamoto
Local IRB – Japan Site 040	Fukuoka University Hospital IRB 7-45-1,Nanakuma, Jonan-ku, Fukuoka-shi, Fukuoka 814-0180,Japan	Shinichi Imafuku
Local IRB – Japan Site 047	Akita University Hospital IRB 44-2, Hasunuma, Hiroomote, Akita-shi, Akita 010-8543, Japan	Katsunori Iijima
Local IRB – Japan Site 051	lRB of Showa University Karasuyama Hospital 6-11-11, Kitakarasuyama, Setagaya-ku, Tokyo 157-8577, Japan	Takemi Yoshida
Local IRB – Japan Site 054	Tokyo Metropolitan Matsuzawa Hospital IRB 2-1-1, Kamikitazawa, Setagaya-ku, Tokyo 156-0057, Japan	Hirohiko Harima
Local IRB – Japan Site 055	IRB of Showa University Northern Yokohama Hospital 35-1, Chigasakichuo, Tsuzuki-ku, Yokohma-shi, Kanagawa 224-8503, Japan	Akihiko Kitami
Local IRB – Japan Site 058	Tokushukai Group IRB 1-3-1, Kudanminami, Chiyoda-ku, Tokyo 102-0074, Japan	Suminobu Ito
Local IRB – Japan Site 060	National Hospital Organization Hokuriku Hospital IRB 5963,Nobusue,Nanto-shi, Toyama 939-1893, Japan	Takanori Hashimoto
Central IRB – Japan Site 061	IRB of Toyama Medical Association 336, Ninagawa, Toyama-Shi, Toyama 939-8222, Japan	Tetsuji Sumitani
Local IRB – Japan Site 063	Kansai Rosai Hospital IRB 3-1-69, Inabaso, Amagasaki-shi, Hyogo 660-8511, Japan	Kimihiko Ito
Local IRB – Japan Site 064	Kobe University Hospital IRB 7-5-2, Kusunoki-cho, Chuo-ku, Kobe-shi, Hyogo 650-0017, Japan	Hironobu Minami
Local IRB – Japan Site 066	Hizen Psychiatric Center Institutional Review Board 160, Mitsu, Yoshinogari-cho, Kanzaki-gun, Saga 842-0192, Japan	Ryo Murakawa
Local IRB – Japan Site 070	Sumitomo Hospital Institutional Review Board 5-3-20, Nakanoshima, Kita-ku, Osaka 530-0005, Japan	Katsuhiko Sakaguchi
Local IRB – Japan Site 072	Tokyo Metropolitan Institute for Geriatrics and Gerontology IRB Committee 35-2, Sakae-cho, Itabashi-ku, Tokyo 173-0015, Japan	Kazumasa Harada
Local IRB – Japan Site 078	Saiseikai Yokohamashi Tobu Hospital IRB 3-6-1, Tsurumi-ku, Shimosueyoshi, Yokohama-shi, Kanagawa 230-8765, Japan	Jun Goto
Local IRB – Japan Site 079	Takeda General Hospital Clinical IRB 3-27, Yamaga-machi, Aizuwakamatsu-shi, Fukushima 965-8585, Japan	Koichi Osonoe
Local IRB – Japan Site 081	National Center for Geriatrics and Gerontology IRB 7-430, Morioka-cho, Obu-shi, Aichi 474-8511, Japan	Takanobu Ichino

Until the information herein is released by Otsuka to the public domain, the contents of this document are Otsuka confidential information and should not be duplicated or re-distributed without prior written consent of Otsuka.

Trial Site Number	IEC/IRB Name	IEC/IRB Chair Name
Local IRB – Japan Site 085	Saga University Hospital IRB 5-1-1, Nabeshima, Saga-shi, Saga 849-8501, Japan	Masatoshi Yokoyama
Local IRB – Japan Site 087	Jinseikai Sugiyama Hospital IRB 41, Kitanodetomichiwaki, Showaokubo, Katagami-shi, Akita 018-1401, Japan	Syuuta Watanabe
Local IRB – Japan Site 096	IRB, Ehime University Hospital Shitsukawa, Toon, Ehime 791-0295, Japan	Koji Sayama
Local IRB – Japan Site 099	General Hanamaki Hospital IRB 4-56, Otaya-cho, Hanamaki-shi, Iwate 025-0082, Japan	Masashi Sawada
Local IRB – Japan Site 101	lRB of Sanyukai Abe Clinic 6-60-10, Higashinippori, Arakawa-ku, Tokyo 116-0014, Japan	Nobuhide Hirai
Local IRB – Japan Site 102	Osaka Psychiatric Medical Center IRB 3-16-21, Miyanosaka, Hirakata-shi, Osaka 573-0022, Japan	Shuya Nishikura
Local IRB – Japan Site 106	Yokohama General Hospital IRB 2201-5, Kurogane-cho, Aoba-ku, Yokohama-shi, Kanagawa 225-0025, Japan	Ken Nagata
Local IRB – Japan Site 108	National Hospital Organization Maizuru Medical Center IRB 2410, Azayukinaga, Maizuru-shi, Kyoto 625-8502, Japan	Nobutaka Ayani
Local IRB – Japan Site 114	Seimou Hospital Institutional Review Board 559-1, Kanohara, Tomioka-shi, Gunma 370-2455, Japan	Chihiro Takeda
Local IRB – Japan Site 116	Japanese Red Cross Akita Hospital IRB 222-1, Kamikitatesaruta Aza Nawashirosawa, Akita-shi, Akita 010-1495, Japan	Masahiko Murata
Local IRB – Japan Site 118	Yamagata University Hospital IRB 2-2-2 Iida-nishi, Yamagata-shi, Yamagata 990-9585, Japan	Yasuyuki Ota
Central IRB – Japan Site 119	Oita Oka Hospital IRB 3-7-11, Nishitsurusaki, Oita-shi, Oita 870-0192, Japan	Shigetaka Yanagisawa
Local IRB – Japan Site 120	Minamitoyama Nakagawa Hospital IRB 146, Oomachi, Toyama-shi, Toyama 939-8073, Japan	Keiko Miyazu
Central IRB – Japan Site 124	Saitama Medical University Hospital IRB 38, Morohongo, Moroyama-machi, Iruma-gun, Saitama 350-0495, Japan	Keiji Yamamoto
Central IRB – Japan Site 125	Zenshukai Hospital IRB 54-1, Utsuboimachi, Maebashi-shi, Gunma 379-2115, Japan	Masashi Kimura
Central IRB – Japan Site 128	Yoga Allergy Clinic IRB 4-32-16, Yoga, Setagaya-ku, Tokyo 158-0097, Japan	Toshikazu Nagakura
Central IRB – Japan Site 136	Suda Clinic IRB 2-8-14, Takadanobaba, Shinjuku-ku, Tokyo 169-0075, Japan	Tetsuo Hosoya
Local IRB – Japan Site 139	Iwate Prefectural Hospital Central IRB 1-4-1, Ueda, Morioka-shi, Iwate 020-0066, Japan	Yasuaki Katsumata
Central IRB – Japan Site 141	Aida Hospital IRB	Motohiko Aida

Until the information herein is released by Otsuka to the public domain, the contents of this document are Otsuka confidential information and should not be duplicated or re-distributed without prior written consent of Otsuka.

Trial Site Number	IEC/IRB Name	IEC/IRB Chair Name
	216, Motomachi,Yabuki-machi, Nishishirakawa-gun, Fukushima 969-0213, Japan	
Central IRB – Japan Sites 001,048,113,117,129,133	Non-Profit Organization MINS IRB<br>5-20-9-401, Mita, Minato-ku, Tokyo 108-0073, Japan	Akihiko Yura
Central IRB – Japan Sites 002, 049, 050	Jisenkai Nanko Psychiatric IRB<br>33, Sekibehikimehashi, Shirakawa-shi, Fukushima 961-0021, Japan	Kiyoshi Ariga
Central IRB – Japan Sites 003, 019, 023, 088, 121	Mizuo Clinic IRB<br>3-9-1, Nakataminami, Izumi-ku, Yokohama-shi, Kanagawa 245-0014, Japan	Toshiki Mano
Central IRB – Japan Sites 005, 012, 017, 031, 043, 044, 046, 053, 067, 075, 086, 093, 110, 111, 122, 130, 142	Medical Corporation TOUKEIKAI Kitamachi Clinic IRB<br>1-1-3, Kichijoji-kitamachi, Musashino-shi, Tokyo 180-0001, Japan	Hiroshi Ohta
Central IRB – Japan Sites 007, 010, 013, 041	Ekihigashi Dermatology・Allergy Clinic<br>1-12-6, Hakataekihigashi, Hakata-ku, Fukuoka-shi, Fukuoka 812-0013, Japan	Juichiro Nakayama
Central IRB – Japan Sites 008, 039, 042, 065	Goshogatani Home Clinic IRB<br>1-6-1 Maizuru, Chuo-ku, Fukuoka-shi, Fukuoka 810-0073, Japan	Kensuke Sasaki
Central IRB – Japan Sites 009, 137	Haradoi Hospital IRB<br>6-40-8, Aoba, Higashi-ku, Fukuoka-shi, Fukuoka 813-8588, Japan	Samon Koyanagi
Central IRB – Japan Sites 018, 056, 094	Maebashi Hirosegawa Clinic IRB<br>2-10-9, Chiyodamachi, Maebashi-shi, Gunma 371-0022, Japan	Shoji Okamoto
Central IRB – Japan Sites 022, 025, 026, 052, 057, 073, 084, 109, 127, 134	Yoyogi Mental Clinic IRB<br>4-26-11, Sendagaya, Shibuya-ku, Tokyo 151-0051, Japan	Soichiro Watanabe
Central IRB – Japan Sites 027, 030, 045, 059, 071, 074, 077, 080, 082, 090, 091, 092, 098, 100, 123, 140	Sugiura Clinic IRB<br>4-4-16-301, Hon-cho, Kawaguchi-shi, Saitama 332-0012, Japan	Kazuhiko Watabe
Central IRB – Japan Sites 033, 076	Osaka Institute of Clinical Psychiatry Shin-abuyama Hospital IRB<br>4-10-1, Nasahara, Takatsuki-shi, Osaka 569-1041, Japan	Tetsuya Yoshimoto
Central IRB – Japan Sites 036, 083, 097, 104, 132, 138	Adachikyousai Hospital IRB<br>1-36-8, Yanagihara, Adachi-ku, Tokyo 120-0022, Japan	Masahisa Nakamoto
Central IRB – Japan Sites 062, 115	Review Board of Human Rights and Ethics for Clinical Studies IRB	Norihiko Basugi

Until the information herein is released by Otsuka to the public domain, the contents of this document are Otsuka confidential information and should not be duplicated or re-distributed without prior written consent of Otsuka.

**Trial Site Number**	**IEC/IRB Name**	**IEC/IRB Chair Name**
	2-2-1, Kyobashi, Chuo-ku, Tokyo 104-0031, Japan	
Central IRB – Japan Sites 068, 069	Keishinkai Kyowa Hospital IRB<br>277-1, Gohyakukarida, Kyowakamiyodokawa, Daisen-shi, Akita 019-2413, Japan	Yukiko Mishima
Central IRB – Japan Sites 089, 105, 112, 126	Mental Clinic Sakurazaka Clinical Trial Review Committee<br>3-6-1, Kego, Chuo-ku, Fukuoka-shi, Fukuoka 810-0023, Japan	Tetsuji Matsuyoshi

Until the information herein is released by Otsuka to the public domain, the contents of this document are Otsuka confidential information and should not be duplicated or re-distributed without prior written consent of Otsuka.
